# Supplementary material for: Do lifestyle factors affect patient reported clinical outcomes after total knee replacement surgery? A feasibility cohort study (PRO-Knee)
Source: PLoS One. 2025 Oct 21;20(10):e0332953. doi: 10.1371/journal.pone.0332953 (PMC12539706; doi:10.1371/journal.pone.0332953)
Supplement: S1 Table — (DOCX) [file pone.0332953.s001.docx]

**Supporting Information**

**S1 Table.**

**Management of Patient reported outcomes data.**

| **Variable and measurement tool** | **Scoring** | **Missing data** |
| --- | --- | --- |
| Awareness of operated knee  Forgotten Joint Score | 12 questions providing an overall score between 0-100 | Outcome measure withdrawn if >3 missing values. |
| Knee pain and function  Oxford Knee Score | 12 questions providing an overall score between 0-48 | Sub-scale mean to be used if <3 missing values.  Outcome measure withdrawn if >2 missing values. |
| Health Related Quality of Life  EQ5D5L Index | All values were indexed. −0.594 (worst state) to 1.0 (best state). | Multiple imputation to be used for missing values. |
| Anxiety  Hospital Anxiety and Depression Scale (HADS) | 0-21 | Sub-scale mean used for missing values providing > 50% of answers provided. |
| Depression  Hospital Anxiety and Depression Scale (HADS) | 0-21 | Sub-scale mean used providing that more 50% of answers in total were provided |
