# Supplementary material for: Do lifestyle factors affect patient reported clinical outcomes after total knee replacement surgery? A feasibility cohort study (PRO-Knee)
Source: PLoS One. 2025 Oct 21;20(10):e0332953. doi: 10.1371/journal.pone.0332953 (PMC12539706; doi:10.1371/journal.pone.0332953)
Supplement: S2 Table — (DOCX) [file pone.0332953.s002.docx]

**S2 Table.**

**Number of lifestyle factors and clinical outcomes for all participants**

|  | **Forgotten Joint Score** | | | **Oxford Knee score** | | |
| --- | --- | --- | --- | --- | --- | --- |
| **No of lifestyle factors** | **Baseline** | **3-months** | **6-months** | **Baseline** | **3-months** | **6-months** |
| **1 Lifestyle factor**  Number of participants (%)  Mean score (SD) | 13 (100)  87.5 (7.8) | 13 (100)  64.6 (31.0) | 11 (84.6)  61.1 (26.1) | 13 (100)  20.5 (7.5) | 12 (92.3)  29.7 (11.0) | 11 (84.6)  33.9 (7.3) |
| **2 Lifestyle factors**  Number of participants (%)  Mean score (SD) | 21 (100)  91.5 (8.3) | 18 (85.7)  78.1 (19.4) | 17 (81.0)  78.5 (18.6) | 21 (100)  16.9 (7.4) | 18 (85.7)  25.2 (8.50 | 17 (81.0)  26.3 (9.5) |
| **3 Lifestyle factors**  Number of participants (%)  Mean score (SD) | 6 (100)  87.2 (12.2) | 6 (100)  77.9 (27.3) | 6 (100)  61.0 (36.6) | 6 (100)  18.7 (8.0) | 6 (100)  27.8 (12.2) | 6 (100)  30.5 (13.9) |
