# Supplementary material for: Do lifestyle factors affect patient reported clinical outcomes after total knee replacement surgery? A feasibility cohort study (PRO-Knee)
Source: PLoS One. 2025 Oct 21;20(10):e0332953. doi: 10.1371/journal.pone.0332953 (PMC12539706; doi:10.1371/journal.pone.0332953)
Supplement: S3 Table — (DOCX) [file pone.0332953.s003.docx]

**S3 Table**

**Satisfaction with aspects of outcome at 6-months post total knee replacement**

| **How satisfied are you with…** | **Very dissatisfied**  **(%)** | **Dissatisfied**  **(%)** | **Neither satisfied nor dissatisfied (%)** | **Satisfied**  **(%)** | **Very satisfied**  **(%)** | **Missing data (%)** |
| --- | --- | --- | --- | --- | --- | --- |
| the changes you have experienced in your pain due to your knee replacement? | 2/35 (5.7) | 3/35 (8.6) | 7/35 (20.0) | 10/35 (28.6) | 13/35 (37.1) | 5/40 (12.5) |
| the changes you have experienced in your walking due to your knee replacement? | 2/35 (5.7) | 4/35 (11.4) | 9/35 (25.6) | 7/35 (20.0) | 13/35 (37.1) | 5/40 (12.5) |
| the appearance of your knee replacement? | 1/35 (2.8) | 3/35 (8.6) | 2/35 (5.7) | 15/35 (42.9) | 14/35 (40) | 5/40 (12.5) |
| the changes you have experienced in your ability to take part in hobbies / leisure activities | 3/34 (8.8) | 3/34 (8.8) | 13/34 (38.2) | 6/34 (17.6) | 9/34 (26.4) | 6/40 (15.0) |
| the outcome of your knee replacement at this point? | 3/35 (8.6) | 5/35 (14.3) | 7/35 (20.0) | 8/35 (22.9) | 12/35 (34.3) | 5/40 (12.5) |

Key - Number of responses / Total responses
