# Supplementary material for: Do lifestyle factors affect patient reported clinical outcomes after total knee replacement surgery? A feasibility cohort study (PRO-Knee)
Source: PLoS One. 2025 Oct 21;20(10):e0332953. doi: 10.1371/journal.pone.0332953 (PMC12539706; doi:10.1371/journal.pone.0332953)
Supplement: Attachment — Submitted filename: PLOS One reviewer feedback_21 MAY 25.docx [file pone.0332953.s005.docx]

**Reviewer Comments to the Author**

**Reviewer #1: Thank you very much for allowing me to review your manuscript. I appreciate the effort you have made performing this study and submitting it to PLOS one.
From my prospective, the sample size is low and it can result in different error in final conclusion. So, the biggest sample size is warranted.**

Thank you for your feedback. This study is a feasibility cohort study which aims to determine whether a substantive cohort study is feasible to deliver. We accept that the study is not adequately powered to make definitive conclusions about whether lifestyle factors affect outcomes from total knee replacement surgery. This is why the results focus on pre-defined feasibility outcomes. In this context, the target sample size was 40 participants which is above the median (n=30) and on the upper end of the IQR (n=20-43) of recent (2013-2020) UK pilot and feasibility studies with continuous outcomes, on the ISRCTN registry. We therefore feel that the sample size is adequate in relation to the study objectives.

Reviewer #2: Comments on a manuscript submitted to PLOS One entitled:

**Do Lifestyle Factors affect patient-reported clinical outcomes after total knee
replacement surgery? A feasibility cohort study (PRO-Knee)
(# PONE-D-25-04937)

General Comment
Thank you for the opportunity to review the above-mentioned submission, which evaluates the feasibility of a large-scale study into whether lifestyle factors affect patient-reported clinical outcomes following a total knee replacement (TKA). If and how different modifiable lifestyle factors affect the outcomes following TKA is a very important question, and I appreciate the author’s efforts to tackle this topic. However, as it stands now, the manuscript needs some revisions before it is suitable for publication. Below, I have provided some comments that may assist the authors in enhancing the presentation of their work.**

Thank you for your feedback, we have addressed each of your suggested revisions in the comments below and reference to the changes have been made in the revised manuscript.

**Specific comments
Introduction
p. 3 Line 70: A TKA aims to relieve pain and restore function/ADL ability and, ultimately, patients’ quality of life. I suggest adding that information.**

Line 70 – ‘(pain, function and quality of life)’ has been added to the manuscript to make it clear which domains are commonly measured by clinical outcomes.

**p. 3 Line 81: Please introduce abbreviations before using them.**

Lines 77-78 – ‘NICE’ has been amended to ‘National Institute of Health and Care Excellence’

**p. 3; Line 83: Again: Please introduce abbreviations before using them.**

Line 80 – ‘UK NHS’ has been changed to ‘United Kingdom National Health Service’.

**p. 3; Lines 91/92: I do not fully agree with this sentence. Although it may not be fully understood, there is evidence to suggest an association between lifestyle factors and inferior clinical outcomes following total joint replacement. I suggest rephrasing that statement to read less strongly.**

Line 89 – ‘to what extent’ has been added

**Methods
p. 5; Line 138: You referred to Table 1 already in Line 136. So, no need to refer to it here again. In fact, I suggest combining the sentences lines 135/136 and 138/139 to avoid redundancies.**

Line 134-137 – The sentences have been combined to read ‘Patients who provided consent but did not meet the enrolment criteria did not proceed in the study as they did not demonstrate one of the lifestyle factors of interest using validated screening tools (13-15)’

**pp. 5 & 6: It may be due to the formatting in the submission portal, but please make sure not to have dangling tables in the manuscript.**

This occurs due to the submission portal, none of the tables cross pages in the submitted text. This may change again if tracked changes are accepted. To be clear – the line numbers refer to the document with tracked changes in (that have not been accepted) so that you can easily see the changes made. If tracked changes are accepted the formatting of the document will change again and the line referencing in this document will not be synchronised.

**p. 6; Table 1: Please add a caption for BMI.**

Line 145 - The caption has been added

**p. 6, Lines 158 and 164: Five instead of 5.**

Lines 153, 157 & 159 – ‘5’ has been changed to ‘five’

**p. 6/7: Please provide data on the validity and reliability of the AUDIT C, GPPAQ, and Functional Comorbidity Index for your population.**

The AUDIT C, and Functional Comorbidity Index are screening tools used to identify alcohol consumption and co-morbidities. We do not therefore believe that it would be expected that these screening tools would be validated in a population undergoing total knee replacement.

Line 164-165 – The following statement has been added. ‘Whilst not validated in a total knee replacement population, it is recommended by NICE as a screening tool to measure physical activity’.

**p. 7; Line 176: What exactly covers “functional status”? Only musculoskeletal physical functioning, general physical functioning, or even cognitive aspects? Was the Functional Comorbidity Index (FCI) collected only for this study, or is routinely collected data available from the patient’s medical record? If it was collected outside the clinical routine specifically for this study, why was it important to have that data? You do not report it other than as a mean of comorbidities in the subject characteristics table.**

Line 172 – ‘physical function’ has been added to make this clearer. The tool was used to report mean number of comorbidities in this feasibility study.

Line 173-174 – ‘which could be used as part of secondary analysis of a future substantive study’ has been added to describe how the tool could be used as part of a future substantive cohort study.

**p. 7; Line 177: When pre-operatively? On the day of admission? Or earlier? Was that time the same for all subjects?**

Line 176 – ‘(within 12-weeks of surgery)’ has been added.

**p. 7; Line 178: Figure instead of Fig.**

Line 177 – changed as requested.

**p. 7; Line 181 to p. 8; Line 205: Please provide data on the validity and reliability of the used PROMS (FJS, OKS, EQ5D5L, and HADS).**

Lines 185-188, 193-194, 203-205 and 211-213 have been amended to add this reliability / validity data

**p. 7; Line 185: Why did you choose to use the OKS if you already knew it demonstrates a larger ceiling effect in your investigated patient population? There are a lot of other knee-specific PROMs available in the English language.**

The OKS was included as a secondary outcome measure as it is the most common patient-reported measure of pain and disability in total knee replacement trials. The Forgotten Joint Score used as the primary outcome measure, as it evaluates how aware individuals are of the knee in daily life and demonstrated a higher ceiling effect.

**p. 9; Line 220: Please introduce the abbreviation ISRCTN**

Line 248 – ‘International Standard Randomised Controlled Trial Number’ has been added.

**p. 9; Line 223: “Date are” instead of “Data is,” as data are always plural.**

Line 252 – change made as requested.

**p. 9; Line 225: What was your preset significance level? Which software did you use for the statistical analysis? What about those with two lifestyle factors? E.g., the 7.5% alcohol & high BMI or the 42.5% high BMI and Physical inactivity? Did you not include them in your analysis? Please revise accordingly.**

Line 256 – The significance level (p=<0.05) has been added.

As this was feasibility cohort study that was statistically underpowered to make definitive statements about the relationships between lifestyle factors and clinical outcomes, we have been careful not to make inferences from the data. We felt that comparing those with one lifestyle factor to those with three lifestyle factors would be adequate to test for any signal in the data. We feel that any further statistical analysis risks over-inferring and mis-representing the data.

**p. 9, Table 3: I suggest specifying the table title more. Again, Tables and Figures must be self-explaining and understandable even when taken from their context. In addition, were the different percentages for the success criteria in the red, amber, and green groups chosen arbitrarily or based on a specific (statistical) rationale?**

The title of table 3 has been changed to ‘Success criteria for progression to substantive cohort study’

Line 250-251 – ‘developed using published guidelines’ has been added and referenced.

**Results**
**p. 10; Lines 250/251: Was there a difference in characteristics between those who agreed to participate versus those who declined to participate?**

Thank you for this comment. This data was not recorded or reported as part of the study as these individuals have not provided consent to have their information included as part of the study.

**p. 10/11; Table 4: I suggest completely revising this table to meet scientific reporting standards. In addition, what questions did subjects have to answer regarding their sex/gender? „Male,” as you report, refers to biological sex, whereas „Gender“ is a social construct that should be reported as men/women and may be different from sex at birth. Please be specific in your wording. Moreover, please be consistent in how you list multiple lifestyle factors in your table. Decide whether to use “and” or “&.”**

Thank you for your comments. The table has been revised to meet reporting standards, and the following requested changes have been made:

- The table has been updated so that the use of ‘and’ is consistent.
- Gender has been replaced by ‘sex at birth’ to accurately reflect the question participants were asked.

**Regarding employment status, did your subjects work full-time or half-time, in blue-collar or white-collar jobs? Based on Table 2, you collected this information via questionnaires, so I guess patients were not just asked single-item questions regarding their work status.**

Thank you for your comments. For the purposes of this study, employment status was asked via 1, multiple choice question which asked participants to select an option from:

- Employed
- Self-employed
- Unemployed
- Retired
- Long-term sick or disabled
- Looking after home / family /dependants
- Other

The table therefore accurately reflects the responses of participants. Given the objectives of the study, we did not feel it was required to collect more data than this and we tried to be sensitive to questionnaire burden for participants.

**Regarding co-morbidities, which were those? These factors could influence lifestyle and thus should be considered carefully both in the results and discussion section.**

The functional co-morbidity index reports whether an individual has any of the following co-morbidities: Osteoarthritis, Osteoporosis, Spinal pain, Respiratory conditions, Angina, Myocardial infarction, Heart Failure, Neurological conditions, Dementia, Cerebral Vascular Accident, Peripheral Vascular Disease, Diabetes Mellitus, Gastrointestinal disease, obesity, Depression, Anxiety, Visual or Hearing restriction.

We considered adding this data as part of the original manuscript but decided that it added little value in the context of the objective – to test the feasibility of delivering a future substantive cohort study. The study is interested in whether modifiable lifestyle factors (that may contribute to the development of comorbidities and poor long-term health) affect outcomes from total knee replacement because they are potentially modifiable. The focus on the discussion is therefore on these lifestyle factors, rather than these medical comorbidities. We expect that we would provide more detail on comorbidities as part of a future substantive cohort study as it may add context to the outcomes. However, in this manuscript, we felt that detailed reporting and discussion of comorbidities may distract from the study objectives and could lead to over-inference in a study of 40 participants from one site in the UK.

**p. 11; Table 5: I suggest substantially reworking this table to meet scientific reporting standards. It does not make sense to read “number of participants” under “outcome.” Moreover, measures of central tendency and dispersion are usually reported as “mean (SD)” or “mean (± SD)” instead of one above the other. Furthermore, you should combine the information from Tables 5 and 6 and maybe even Table 7 into one table.**

Table 5 & 6 have been merged and amended in keeping with the suggestions above.

We felt it would be problematic to the reader to also merge with Table 7 has also been amended in line with the comments above.

**p.11; 310: I do not see the EQ5D and HADS scores reported anywhere in the manuscript. What is the reason for that? It seems selective not to report those. Please provide information for these PROMS in the text and the tables.**

The prevalence of anxiety and depression derived from the HADS has been reported in table 2. The sub-heading within the table has been amended to make this clearer.

Lines 412-414 – The following statement has been added to explain why the EQ5D-5L data has not been presented in the manuscript.

‘The Euroqol EQ5D-5L was used to measure health-related quality of life, however NICE (2019) do not recommend the use of this tool until a validated data set for the UK has been established (39)’.

**Discussion
General Comment: I recommend revising the entire discussion substantially. Currently, most parts of the discussion only echo the results section. I do not see the results embedded in the current literature nor really being discussed.**

Thank you for your comments on the discussion section. The feasibility study aims to test whether a larger substantive cohort study is deliverable within the UK NHS.

The discussion adheres to convention in as it opens with a summary of the key findings, then discusses the findings in the context of current evidence base and concludes with the limitations of the study.

We have reviewed the manuscript again amongst the author group and feel that the findings have been discussed within the context of contemporary literature:

The reported rates of lifestyle factors and co-morbidities are high – in keeping with current literature.

The reported dissatisfaction rates from surgery are also high and in keeping with current literature.

Therefore, the main findings of the study are, that a cohort study is necessary and deliverable.

It is tempting to forecast that interventions which target unhealthy lifestyles may positively influence outcomes from joint replacement surgery and discuss the mechanisms by how they may work. However, given that this is a feasibility study and no definitive relationship between lifestyle factors and clinical outcomes from joint replacement has been established, we feel that any in-depth discussion of this could potentially mis-lead the reader by over-inferring from our sample of 40 participants. We have however incorporated some of the specific suggestions as outlined below and also tried to make the above clearer to the reader.

**Specific comments:
p. 14, Lines 322/323: You state “….suggesting improvements should be considered.” Yet, you do not discuss which ones (other than having more staff available for recruitment) these may be and what your strategies would be to address/implement such “improvements.”**

Lines 354-357 – When the consent rate of patients approached to consent was analysed, the recruitment rate was 30%, which aligned with the green zone in our success criteria. Therefore, we did not make significant recommendations to improve recruitment.

**p. 14, Lines 328/329: Be careful not to make overly strong statements regarding aspects you did not measure. For example, your data do not suggest ‘the prevalence of lifestyle factors associated with poor long-term health in patients undergoing TKA is high.’. You did not analyze associations, did not establish prevalence values, nor did you measure your participants’ health in the long term (neither before nor after the operation). And claiming, based on just one study, which is not even a Systematic Review or Meta-Analysis, that 6 months is a good window to extrapolate outcomes to the long term seems invalid. Furthermore, as you state later, your study was not powered sufficiently to make any conclusions other than, based on your selected criteria, a larger study seems feasible.**

Lines 348-350 – The statement has been softened as outlined below – to reflect the fact that lifestyle factors are associated with long-term poor health, however, our results only suggest that the prevalence of lifestyle factors might be high in people undergoing total knee replacement.

‘The recruitment rate (40/40; 100%) from this feasibility study suggests that the prevalence of lifestyle factors, which are associated with poor long-term health in patients undergoing total knee replacement could be high’.

**p. 14, Line 331: Please add “before surgery” following “physically inactive.” You did not measure it after.**

Lines 351-352 - ‘prior to surgery’ has been added

**In addition, your conclusion is a bit weak, as patients could still be physically active even with knee OA. They could engage in much more than just walking (e.g., aquatic exercises, cycling, fitness training, etc.). In addition, given that subjects had an average of 3.5 comorbidities/long-term health conditions, OA might not be the (only) reason for their physical inactivity. Please discuss this appropriately.**

Thank you for your comments, the discussion does not comment on those who may be active with osteoarthritis. Instead, this study aims to evaluate the prevalence of lifestyle factors in those waiting for total knee replacement. We have included the following statement to reflect the fact that influences on physical inactivity are likely to be multi-dimensional and this would need to be considered as part of any future interventions.

Lines 362-363 - . ‘Future interventions which may target improvement of lifestyle factors such as physical activity would need to consider the multi-dimensional influences on reduced physical activity’.

**p. 14, Line 336: be consistent in your reporting. Your criteria were BMI >24.9**

Line 356 - Thank you, this has been amended.

**p. 14; Line 3343-348: this is all just a repetition of your results.**

Lines 364-367 – This section has been amended to be more concise and lead into a discussion of the satisfaction data in the context of contemporary literature.

**p. 15, Lines 354-356: I would rather say “supports previous findings” instead of “suggests,” as this was already known before this study.**

Line 382 – Thank you, this has been amended.

**p. 15, Lines 373/374: As stated above, I do not think making this assumption based on one single study is valid.**

These points relate to a discussion about the Oxford Knee score and not the data in this study. We have attempted to make sure that the discussion does not over-infer the results of this feasibility study,

**p.16, Lines 377-379: I agree. In addition, there may be differences regarding the socioeconomic and educational status of residents between different regions of the UK, possibly leading to differences in respective patients’ lifestyles and or health status.**

Line 405 **– ‘**due to the diversity of practices and populations across the UK’. Has been added to reflect this point.

**References
Please check your entire reference list for consistency in your citation style. Some journal names are fully written out, while the respective journal’s abbreviation accompanies other references. Some journal names are italicized, others not. Not all references include all necessary information (e.g., #9, where the journal name is missing). Moreover, please provide the last access date with weblinks**.

The reference list and citations within the text have been updated in their entirety.

**Supplement
S1 appendix: Please include information on the number of cases in which the proposed handling of missing data has been needed. Furthermore, here again, health-related quality-of-life data and data for anxiety and depression are mentioned. However, I could not find such data in the results section or the discussion. Please add the EQ5D5L and HADS data to your manuscript and discuss those sufficiently.**

Thank you for your comment – As the purpose of this feasibility study was to determine whether sufficient outcome measures were returned, we have reported this. In line with this and the sample size limitations, this further data was not included and, aligned with the comments and responses above, we feel further discussion would risk over inferring, given the primary objectives of the study. We have tried to emphasise this in the discussion, particularly in lines 374-375.

We have clarified the reason we did not present the EQ5D data as part of our limitations and we feel the HADS data is presented at an appropriate level as part of Table 4.

**S2 appendix: Please re-work the table to meet scientific standards of reporting (e.g., do not use “No” for the number of participants but “N=..” or “n= ..”; report Mean (SD) as 87.5 (SD 7.8) for easier reading; please provide information regarding score interpretation in a table caption to ease data interpretation, etc.)**

All tables in the supplementary information has been updated in line with the recommendations.
