# Supplementary material for: Do lifestyle factors affect patient reported clinical outcomes after total knee replacement surgery? A feasibility cohort study (PRO-Knee)
Source: PLoS One. 2025 Oct 21;20(10):e0332953. doi: 10.1371/journal.pone.0332953 (PMC12539706; doi:10.1371/journal.pone.0332953)
Supplement: Attachment — Submitted filename: PLOS One reviewer feedback_03SEP25.docx [file pone.0332953.s006.docx]

**Response to reviewer comments.**

**Specific comments**

**1. Line 393. It is confusing that “all patients who consented to take part in this study reported at least one relevant lifestyle factor, including smoking tobacco, drinking alcohol, being physically inactive, or overweight”. This means that, based on this feasibility study, the planned multi-center study will not have any control group (patients with no modifiable lifestyle factor) for the comparison. Without a relevant control group, the multi-center study will be meaningless.**

We disagree with this review point and suspect the reviewer has misinterpreted our manuscript and the aims of our feasibility study. Objective 1 clearly states that we wanted to explore ‘The eligibility rate (the number of individuals who met the eligibility criteria as a proportion of those screened).’ It just happens that all those who consented had one or more of the relevant lifestyle factors. This is something we wanted to explore at this feasibility stage and does not mean that a future, definitive study, would not include participants without any of the modifiable lifestyle factors.

**2. Lines 346-348 and 360-363. Patient satisfaction is a complex issue and there are no easy ways to find clinically relevant answers. Therefore, it is unexpected that the current feasibility study was based only on the evaluation of subjective outcome (that is PROMs) and did not include any objective measurements of clinical outcome (such as the measurement of daily physical activity and the measurement of walking speed as an evaluation of functional capacity). The primary outcome measure should be, as proposed, one of the selected PROMs but there must be relevant objective data to characterize the features of dissatisfied patients.**

While we acknowledge the complexity of patient satisfaction, we wish to clarify that our study did not aim to measure satisfaction directly, but instead focused on *clinical outcomes*—specifically pain, function, and quality of life—using validated patient-reported outcome measures (PROMs).

PROMs are widely used in orthopaedic research and clinical practice as reliable and meaningful tools for assessing outcomes that matter most to patients. Therefore we do not agree that objective measurements are required to obtain clinical outcomes for patients, whilst also reducing significant cost and participant burden.

**3. Table I. High BMI is defined as score of > 24.9. Without doubt, obesity is one of the leading factors behind sedentary lifestyle, probably affecting the outcome. However, a slight overweight (such as BMI values of 25-28) hardly are a worrisome risk-factor for middle-aged and older TKR patients due to the natural age-related increase of body weight. Certainly, BMIs of obesity classes certainly are of a concern.**

This review point is speculative, and we confirm that we have adopted the World Health Organization’s classification system, which is the most rigorous and defendable approach we currently have.

**4. Lines 234-238 and 299-302. There was no significant difference in PROMS of those with three lifestyle factors compared with those with one lifestyle factor. However, looking the data of Table 6, those with two lifestyle factors had the worst outcome. If true, is it relevant to analyze the outcome according to the number of different lifestyle factors? Looking the data of Table 4, one option could be to compare patients with high BMI (such as obesity BMIs > 30.0) and physical inactivity to those with non-obesity and non-sedentary lifestyle.**

Whilst it may initially appear that participants with two lifestyle risk factors had worse outcomes than those with three, we believe this is likely due to the small sample size and resultant variability and so there is a risk of over inference if further ‘cherry-picking’ analysis is undertaken.

**5. Lines 358-359. The investigators state that this feasibility study was not powered to make definitive conclusions. Therefore, a future substantive cohort study is indicated. Indeed, the minimum requirement is that the investigators open the planned design of the multi-center cohort study and provide its power analysis based on the data of the current study. How many patients are needed? Is it a plan to compare patients with and without lifestyle factors?**

Again, we disagree with the reviewer on this point, it would be unwise to report the required sample size of a future definitive study at this stage due to the further design decisions that need to be made.

Importantly, this feasibility work has yielded essential data to inform the design of a future multi-centre cohort study. It has provided estimates of the prevalence of key lifestyle factors (smoking, physical inactivity, alcohol consumption, and overweight) and the variability in outcome measures (PROMs). These data will be used to conduct a formal sample size calculation for the larger substantive study in due course.

To clarify, the aim of the future study is not simply to compare patients with and without any lifestyle factors, but rather to examine the association between specific modifiable behaviours and clinical outcomes but we can confirm that patients without any of the lifestyle factors would be eligible to participate.
